# The arms race of ray-finned fish against the derepression of LTR retroelements

**DOI:** 10.1038/s41598-024-81149-9

**Published:** 2024-11-27

**Authors:** Elisa Carotti, Edith Tittarelli, Federica Carducci, Marco Barucca, Adriana Canapa, Maria Assunta Biscotti

**Affiliations:** 1 Dipartimento di Scienze della Vita e dell’Ambiente, Università Politecnica delle Marche, Via Brecce Bianche, Ancona, 60131 Italy; 2 Scuola Universitaria Superiore Pavia – IUSS, Piazza della Vittoria n.15, Pavia, 27100 Italy

**Keywords:** Gene expression, Development, Transcriptomics

## Abstract

Transposable elements (TEs) are dynamic components of eukaryotic genomes that are important in speciation. Evolutionary pressure has led to the evolution of silencing mechanisms, such as the involvement of Krüppel box-associated zinc finger proteins (KRAB-ZFPs) that regulate TE transcription via TRIM28-mediated NuRD complex recruitment in sarcopterygians. The transcriptional activity of genes that encode proteins of the NuRD complex was also detected in actinopterygians, even though genes encoding crucial proteins such as TRIM28 and KRAB-ZNF are missing in this evolutionary lineage. This study hypothesized that TRIM33 could serve as a replacement for TRIM28 in this lineage and suggested an evolutionary relationship between the sarcopterygian KRAB-ZFPs, the actinopterygian KRAB-like ZNFs and the fish N-terminal zinc-associated domain (FiNZ) ZNFs that are specific to cyprinids. Through coimmunoprecipitation analyses conducted in the basal teleost *Anguilla marmorata* and the cyprinid *Danio rerio*, we evaluated the interaction of TRIM33 with the KRAB-like and FiNZ ZFPs. Moreover, we explored the expression profiles of TEs and genes that encode proteins that are potentially involved in their silencing during zebrafish development. Our results may provide new evidence for more thoroughly understanding the evolutionary mechanisms underlying the adaptation of ray-finned fish.

**Supplementary Information:**

The online version contains supplementary material available at 10.1038/s41598-024-81149-9.

## Introduction

Transposable elements (TEs) are dynamic components of eukaryotic genomes and are considered to be among the main drivers of speciation. Owing to their ability to transpose from one genomic location to another, silencing mechanisms have evolved to counteract possible deleterious consequences for the genome^1^. An increasing number of papers are challenging the general view that TEs are harmful elements, highlighting that they can be involved in rewiring the gene expression network^2^ and can act in a tissue-specific manner^3,4^. In sarcopterygians (also known as lobe-finned fish, which include coelacanths, lungfishes, and tetrapods), TE transcription is regulated by the interaction with Krüppel-associated box (KRAB) zinc finger (ZNF) proteins (KRAB-ZFPs), which bind the TE sequence with the ZNF motifs located at the C-terminus and recruit tripartite motif protein 28 (TRIM28, also known as KRAB-Associated Protein 1 (KAP1)) with the KRAB domain located at the N-terminus^5–10^. TRIM28 acts as a scaffold for the recruitment of heterochromatin protein 1 (HP1), DNA methyltransferases (DNMTs), and proteins of the nucleosome remodelling deacetylase complex (NuRD), which contribute to the deposition of repressive epigenetic modifications at both the DNA and histone levels to increase the heterochromatin status^11–14^. In actinopterygians (known as ray-finned fishes, including bichirs, sturgeons, gars, bowfins, and teleosts), our previous studies revealed that genes encoding these proteins are transcriptionally active when TEs are transcribed^3,15^. However, in the evolutionary lineage of ray-finned fishes, both the *TRIM28* and *KRAB*-*ZNF* genes are absent. By reconstituting the phylogeny of the TRIM family in actinopterygians, we hypothesized that TRIM33 could act as a possible substitute for TRIM28 in fish and further identified another group of ZNF proteins with a KRAB-like domain. The interaction between the KRAB-like domain and TRIM33 was investigated by docking simulation^15^. Recently, Wells and colleagues^16^ reported the identification of *ZNF* genes with a conserved fish N-terminal zinc finger-associated (FiNZ) domain, which is uniquely and abundantly distributed in cyprinids. These authors demonstrated that FiNZ-ZNFs (FZNFs) diversified following TE expansion and are involved in the repression of TEs during early embryogenesis in zebrafish, which is similar to the function of KRAB-ZNF proteins in tetrapods.

On the basis of these assumptions, the interaction of TRIM33 with KRAB-like ZNF and FZNF proteins was evaluated in vitro via coimmunoprecipitation analyses of the basal teleost *Anguilla marmorata* and the cyprinid *Danio rerio*. The evolutionary relationships of KRAB-like ZNF and FZNF proteins with sarcopterygian KRAB-ZFPs were investigated through phylogenetic analysis. Moreover, we evaluated the transcriptional profile of TEs as well as that of genes potentially involved in silencing mechanisms during zebrafish embryonic development and in early embryos exposed to 5-aza-2’-deoxycytidine (5-aza-dC), a DNA demethylating agent known to derepress TEs^17^.

## Results and discussion

### Interaction of TRIM33 with KRAB-like ZNF and FiNZ ZNF proteins

TRIM28 is a member of the TRIpartite Motif (TRIM) family and has a conserved N-terminal Really Interesting New Gene (RING) E3 ubiquitin ligase domain, two B-box domains, one antiparallel coiled-coil (CC) domain (these three domains are named RBCC), an intrinsically disordered region (IDR), and a C-terminal tandem plant homology-bromodomain (PHD-BD). Among members of the TRIM family, this domain structure is shared by a subset of proteins called Transcription Intermediary Factor 1 (TIF1). In addition to TRIM28, this protein family includes TRIM24, TRIM33, and TRIM66^18^. In contrast to TRIM24 and TRIM66, TRIM33 is distributed among Actinopterygii and has a well-conserved RBCC-IDR-PHD-BD structure^19^. Moreover, the predicted 3D structure of *D. rerio* TRIM33 is superimposable with that of TRIM28 from *Homo sapiens*, as characterized by NMR and X-ray crystallography^20,21^ (Supplementary Fig. S1). The K296/M297/L300 residues present in the CC region of TRIM28 are involved in the interaction with the KRAB domain^20,21^. Similarly, in TRIM33, we suggest that K373 and I374/F377 (both hydrophobic, such as M297/L300) interact with the KRAB-like domain^15^. Therefore, TRIM33 could work as a substitute for TRIM28, which could be recruited to TE genomic loci in fish.

**Fig. 1:**
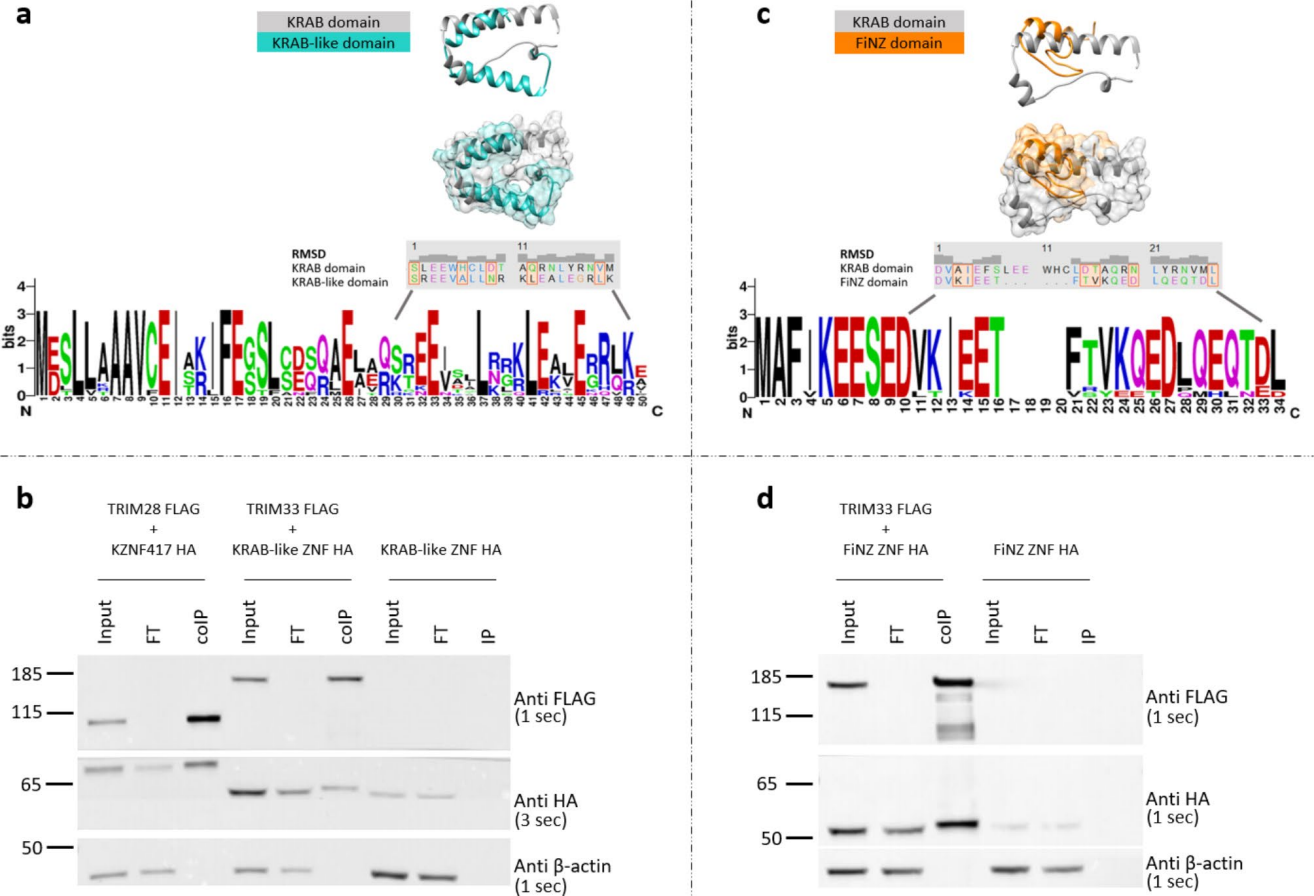
Comparison between KRAB domain and KRAB-like/FiNZ domains and interaction of TRIM33 with KRAB-like ZNF and FiNZ ZNF proteins. (**a**) In the upper side the superimposition between the deposited KRAB domain structure of human KZNF93 and the predicted structure of *Anguilla* KRAB-like domain is reported. In the bottom side the residues of human KRAB domain and eel KRAB-like domain having the best match are aligned and correspond to the position 30–49 of eel KRAB-like ZNF protein, as showed in the logo generated by WebLogo from the multiple alignment of KRAB-like domains. RMSD (*Root Mean Square Deviation*) is an indicator of variability when highly similar proteins are compared. (**b**) From left to right the co-IP assay of human TRIM28 and KZNF417, eel TRIM33 and KRAB-like ZNF, and KRAB-like ZNF negative control. Time of exposure is reported in brackets. (**c**) In the upper side the superimposition between the deposited KRAB domain structure of human KZNF93 and the predicted structure of *D. rerio* FiNZ domain is reported. In the bottom side the residues of human KRAB domain and cyprinid FiNZ domain having the best match are aligned and correspond to the position 10–34 of zebrafish FiNZ ZFP, as showed in the logo generated by WebLogo from the multiple alignment of FiNZ domains. RMSD (*Root Mean Square Deviation*) is an indicator of variability when highly similar proteins are compared. (**d**) From left to right the co-IP assay of zebrafish TRIM33 and FiNZ ZNF and FiNZ ZNF negative control. Time of exposure is reported in brackets. Input = cellular lysate, FT = Flow through, co-IP = co-immunoprecipitated, IP = immunoprecipitate.

The superimposition between the KRAB domain structure of human KZNF93 and the predicted structure of the *Anguilla* KRAB-like domain revealed a significant match between residue positions 30–49 (Fig. 1a). In particular, amino acids V9/I11/F13/L21/V32/M33 of the KRAB domain are hydrophobic and are required for silencing^22^. The KRAB-like domain shares a leucine at position 37 and three other residues at positions 25, 27, and 48, with the same physical properties as those of the KRAB domain (Supplementary Fig. S2). Interestingly, both the glutamic acid residue at position 17 of the KRAB domain and the residue at position 33 of KRAB-like domain were present. Indeed, this residue, if mutated, inhibits binding to TRIM28, which is involved in its silencing activity^22^; thus, it could be crucial for the interaction between the KRAB-like domain and TRIM33.

To validate the formation of a complex involving KRAB-like ZNF and TRIM33, we performed a co-IP assay followed by western blotting and used human TRIM28 and KZNF417 as controls. The results obtained were consistent with a possible interaction between the two proteins, suggesting a TRIM33-dependent function for KRAB-like ZFPs (Fig. 1b).

The structural comparison between the KRAB domain of human KZNF93 and the cyprinid FiNZ domain revealed a match between the residues at positions 10–34 (Fig. 1c). With respect to the hydrophobic amino acids required for silencing, V9 and I11 of the KRAB domain are also present in the FiNZ domain (V11/I13), whereas L21 and Y29 are substituted by residues with similar physical properties (F21/Q29) (Supplementary Fig. S2). Co-IP assays also suggested a possible interaction of FZFPs with TRIM33 (Fig. 1d).

### Expression of *trim33*, *KRAB-like/FiNZ ZNFs*, and transposable elements during zebrafish development

**Fig. 2:**
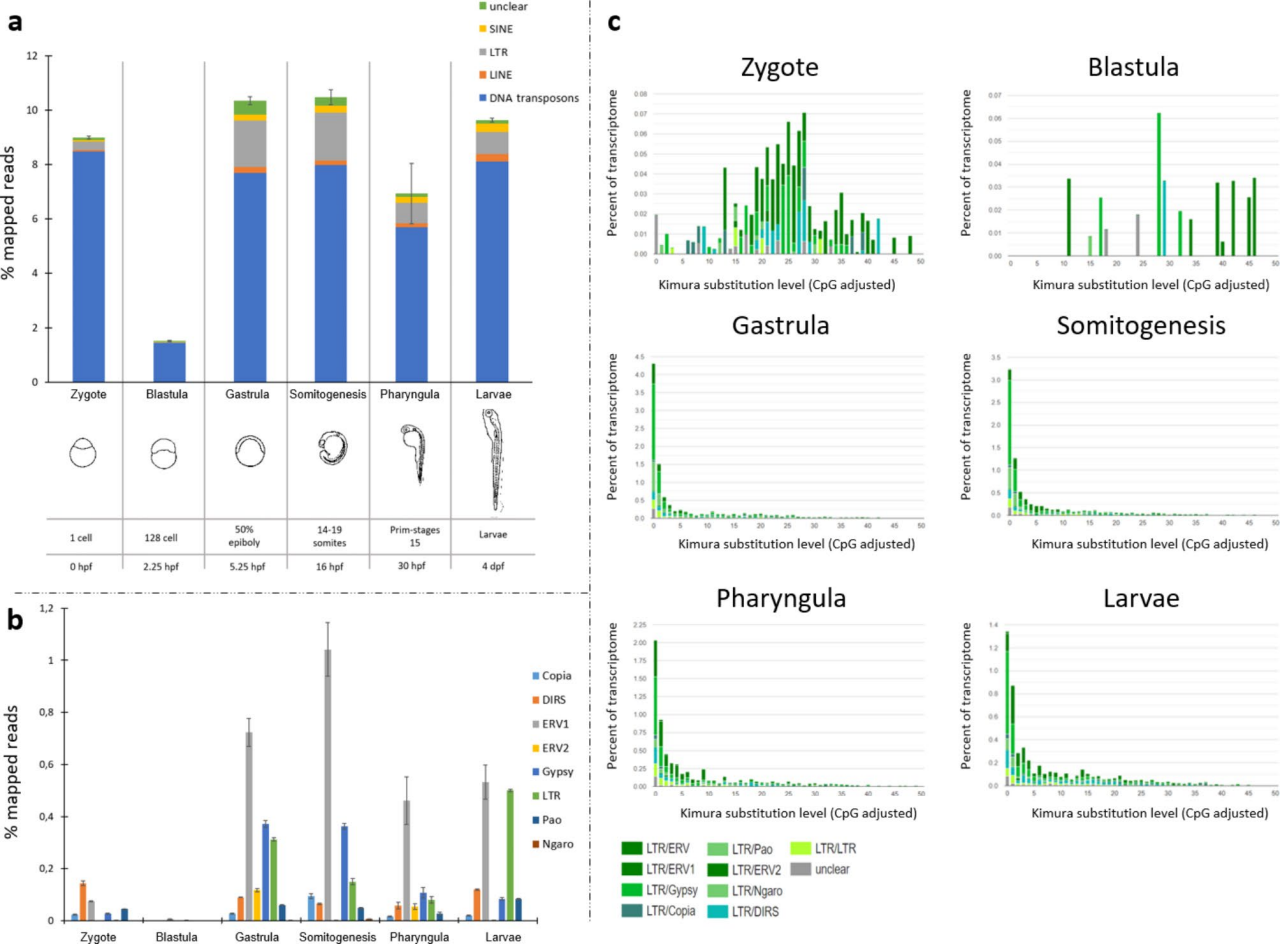
TE transcriptional contribution during zebrafish development. (**a**) Percentage of TE mapped reads in six different zebrafish developmental stages. “Unclear” means TEs that are not classified as DNA transposons, LINE, SINE, and LTR retroelements. (**b**) Percentage of mapped reads related to LTR retroelements in six different zebrafish developmental stages. Elements not classified as Copia, DIRS, ERV1, ERV2, Gypsy, Pao, and Ngaro are included in LTR type. (**c**) The repeat landscape plot obtained by Kimura distance-based copy divergence analyses of LTR retroelements in six different zebrafish developmental stages.

**Fig. 3:**
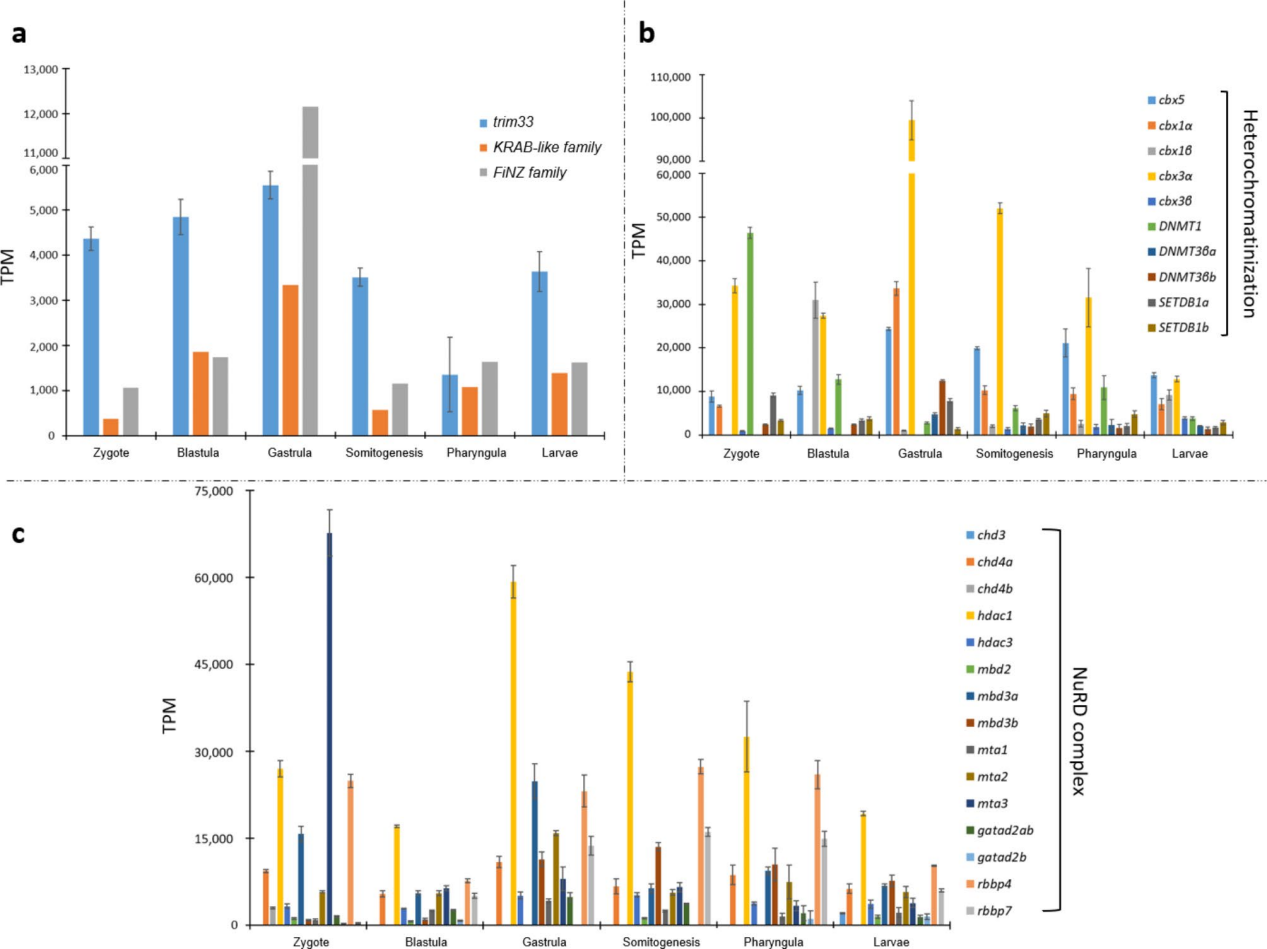
Expression of genes involved in TE silencing mechanisms during zebrafish development. (**a**) Expression values of genes encoding *trim33*, *KRAB-like family* and *FiNZ family* in six different zebrafish developmental stages. We used «family» to indicate that the expression values reported for *KRAB-like* and *FiNZ ZNFs* were an average of values related to different genes. The broken line along the Y axis indicates the range of values from 6,000 to 11,000 TPM. (**b**) Expression values of genes encoding proteins involved in heterochromatin formation in six different zebrafish developmental stages. The broken line along the Y axis indicates the range of values from 60,000 to 90,000 TPM. (**c**) Expression values of genes encoding proteins forming the NuRD complex in six different zebrafish developmental stages.

**Fig. 4:**
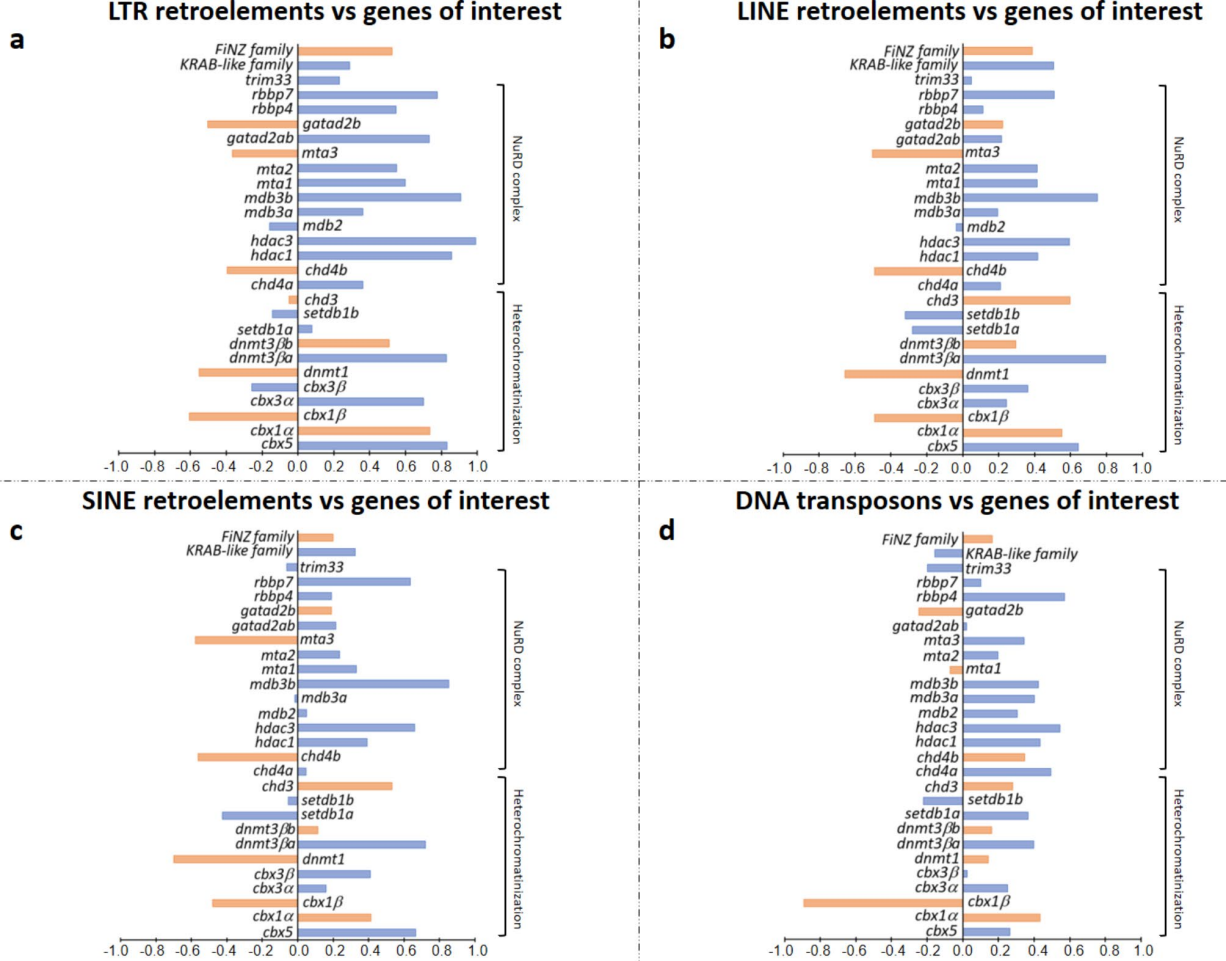
Pearson correlation analyses between TE types and gene expressions during zebrafish development. The bar chart displays the Pearson correlation coefficients, with the horizontal axis representing correlation values and the vertical axis listing individual genes. Blue bars indicate significant correlations, while orange bars indicate correlations not statistically supported. (a) Correlation analysis between LTR retroelements and genes of interest. (b) Correlation analysis between LINE retroelements and genes of interest. (**c**) Correlation analysis between SINE retroelements and genes of interest. (d) Correlation analysis between DNA transposons and genes of interest.

**Fig. 5:**
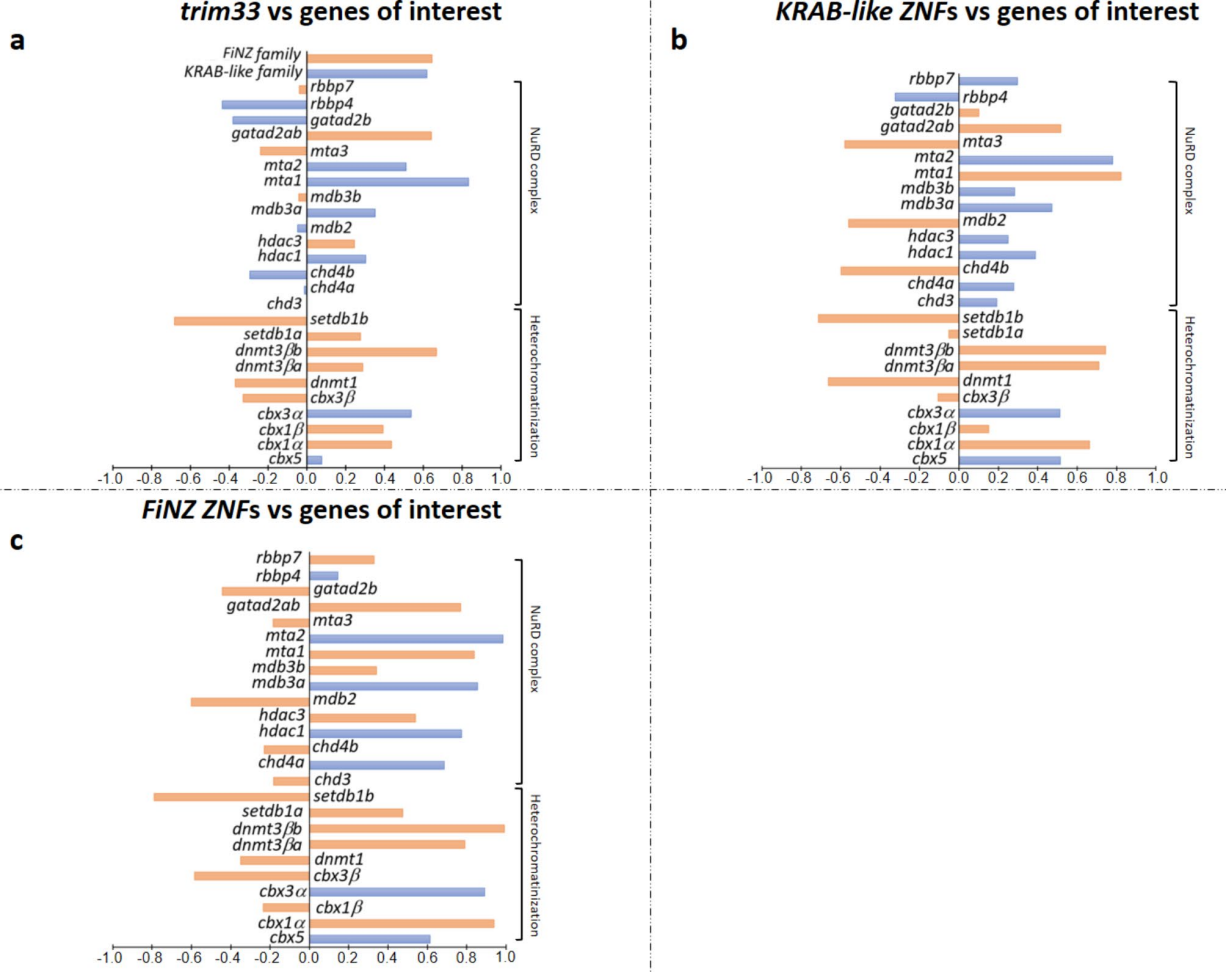
Pearson correlation analyses between TE/gene expressions during zebrafish development. The bar chart displays the Pearson correlation coefficients, with the horizontal axis representing correlation values and the vertical axis listing individual genes. Blue bars indicate significant correlations, while orange bars indicate correlations not statistically supported. (**a**) Correlation analysis between TE expression and genes of interest. (**b**) Correlation analysis between *trim 33* and genes of interest. (**c**) Correlation analysis between *KRAB-like ZNFs* and genes of interest. (**d**) Correlation analysis between *FiNZ ZNFs* and genes of interest.

The involvement of TRIM33 in endogenous retrovirus repression has been demonstrated in mouse testes^23^ and embryonic stem cells^24^. This finding is consistent with its subcellular localization in the nucleus, which was recently reported by Aizaz and colleagues^25^. Margalit and colleagues^24^ proposed a model in which the three members of the TIF1 subfamily form a complex and cooperate to repress retroviral transcription. According to this model, TRIM33 recognizes the acetylated H3 tail of ERVs through its PHD finger-bromodomain, which is a chromatin-binding reader module and functions as a corepressor in support of TRIM28/KRAB-ZFPs. Therefore, it is conceivable that KRAB-like and FiNZ ZFPs bind TRIM33, which in turn acts as TRIM28 to recruit epigenetic silencing proteins to TE loci in fish. Using available RNA-seq data from early zebrafish developmental stages^26^ (Supplementary Table S1), we evaluated the transcriptional profile of TEs (Fig. 2a; Supplementary Fig. S3) as well as the transcriptional activity of genes of interest (Fig. 3). Compared with the other analysed stages, the analysis revealed remarkable TE expression at the 1-cell stage, which is likely attributable to maternally deposited RNA content. These RNA molecules are degraded, and the lower TE activity detected in 128-cell embryos is related to early zygotic genome activation (ZGA). This finding is also consistent with the higher TE transcript levels recorded in the blastula/gastrula stages. Interestingly, the transcriptional enrichment of the LTR superfamily was followed by increased activity of *trim33*, *KRAB-like ZNFs*, and *FZNFs* (Fig. 3a), supporting the hypothesis of their involvement in controlling TEs. The activity of diverse KRAB-like and FiNZ ZFPs (Supplementary Fig. S4) is likely linked to the distinct expression profiles of TE classes and superfamilies during zebrafish development (Fig. 2b), as observed by Chang et al.^27^. The findings shown in Fig. 4 highlighted a significant correlation in the expression between TE types and *KRAB-like ZNFs*. In contrast, the correlation with the *FiNZ ZNFs* was not statistically significant. Therefore, the possible interaction between TRIM33 and FiNZ ZFPs could be associated with other functions that occur during zebrafish development. TRIM33 and KRAB-like ZFPs might silence TEs through the recruitment of heterochromatin proteins (Fig. 3b) and the NuRD complex (Fig. 3c), whose gene expression follows the transcriptional activity profile of TEs. Indeed, the activation of genes encoding proteins that play a role in compacting chromatin, such as heterochromatin proteins (HPs), DNA methyltransferases, and histone deacetylases, was recorded post-ZGA. Moreover, the expression of genes related to the NuRD complex was consistently correlated with the expression of *trim33* and *KRAB-like ZNFs* (Fig. 5). The TRIM28/KRAB ZFPs system represses exogenous retroviruses and their endogenous counterparts LTRs^28–31^. Our results revealed a predominant contribution of ERV1 retroelements during the gastrula, somitogenesis, pharyngula, and larval stages (Fig. 2b). The analysis of TE sequence divergence by Kimura distance performed on the LTR fraction activated during zebrafish development highlighted the presence of recent copies of retroelements (Fig. 2c). Interestingly, the expression of LTR retroelements was more strongly correlated with the genes of the NuRD complex than the expression of DNA transposon, LINE and SINE retroelements was (Fig. 4).

**Fig. 6:**
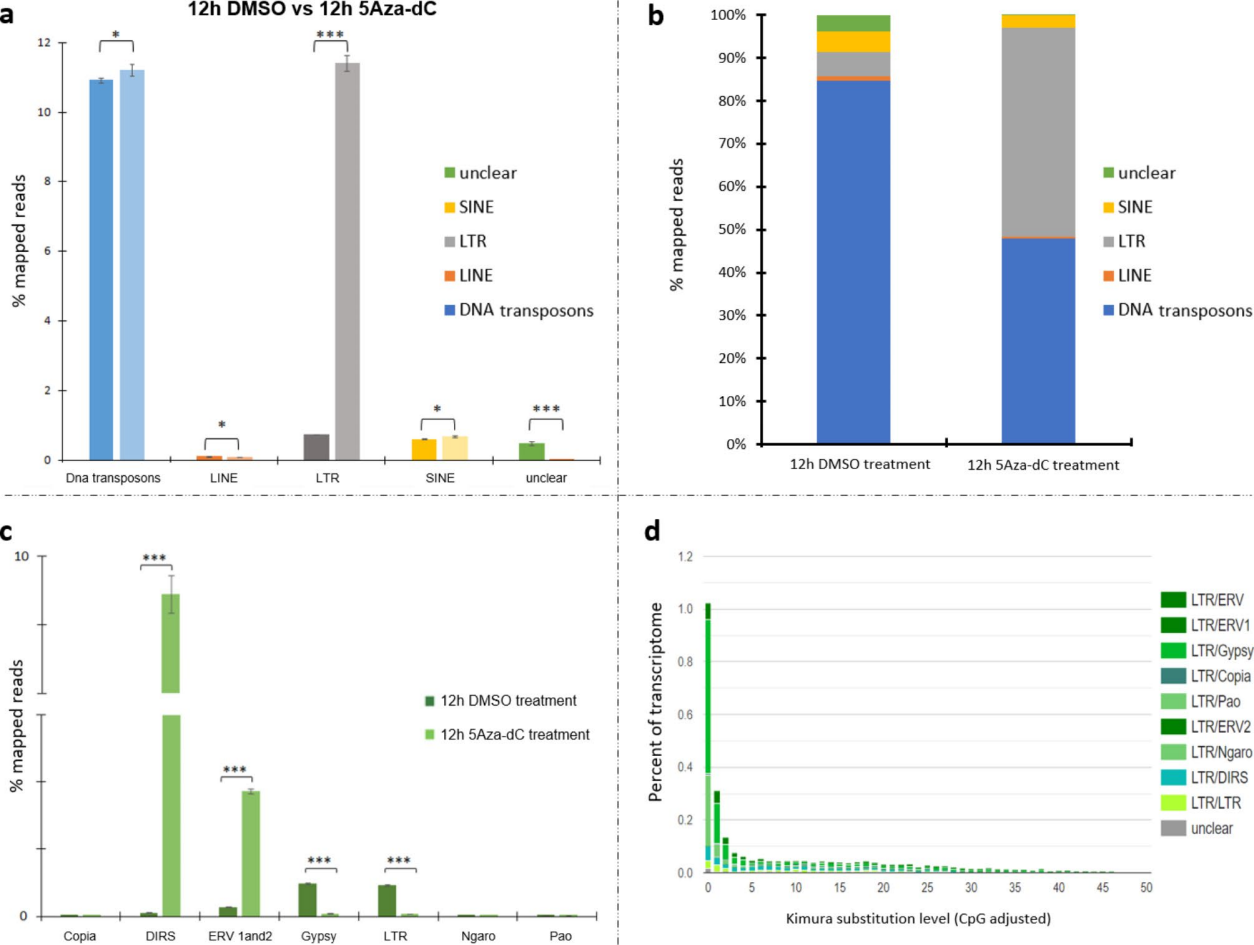
TE transcriptional contribution and LTR repeat landscape plot in zebrafish treated embryos. (**a**) Percentage of TE mapped reads in the RNA-seq data of 12 hpf zebrafish embryos treated with DMSO and 5Aza-dC. In the graph, for each TE class the bar on the left is referred to 12 h DMSO data and that on the right to 12 h 5Aza-dC data. “Unclear” means TEs that are not classified as DNA transposons, LINE, SINE, and LTR retroelements. The statistical analysis was performed using one-way ANOVA. Statistically significant differences are presented as * for *p* < 0.05, ** for *p* < 0.01, and *** for *p* < 0.001. (**b**) Relative abundance of TE mapped reads in the RNA-seq data in the RNA-seq data of 12 hpf zebrafish embryos treated with DMSO and 5Aza-dC. “Unclear” means TEs that are not classified as DNA transposons, LINE, SINE, and LTR retroelements. (**c**) Percentage of mapped reads related to LTR retroelements in the RNA-seq data of 12 hpf zebrafish embryos treated with DMSO and 5Aza-dC. The statistical analysis was performed using one-way ANOVA. Statistically significant differences are presented as * for *p* < 0.05, ** for *p* < 0.01, and *** for *p* < 0.001. The broken line along the Y axis indicates the range of values from 3 to 8% of mapped reads. (**d**) The repeat landscape plot obtained by Kimura distance-based copy divergence analyses of LTR retroelements in the RNA-seq data of 12 hpf zebrafish embryos treated with 5Aza-dC.

**Fig. 7:**
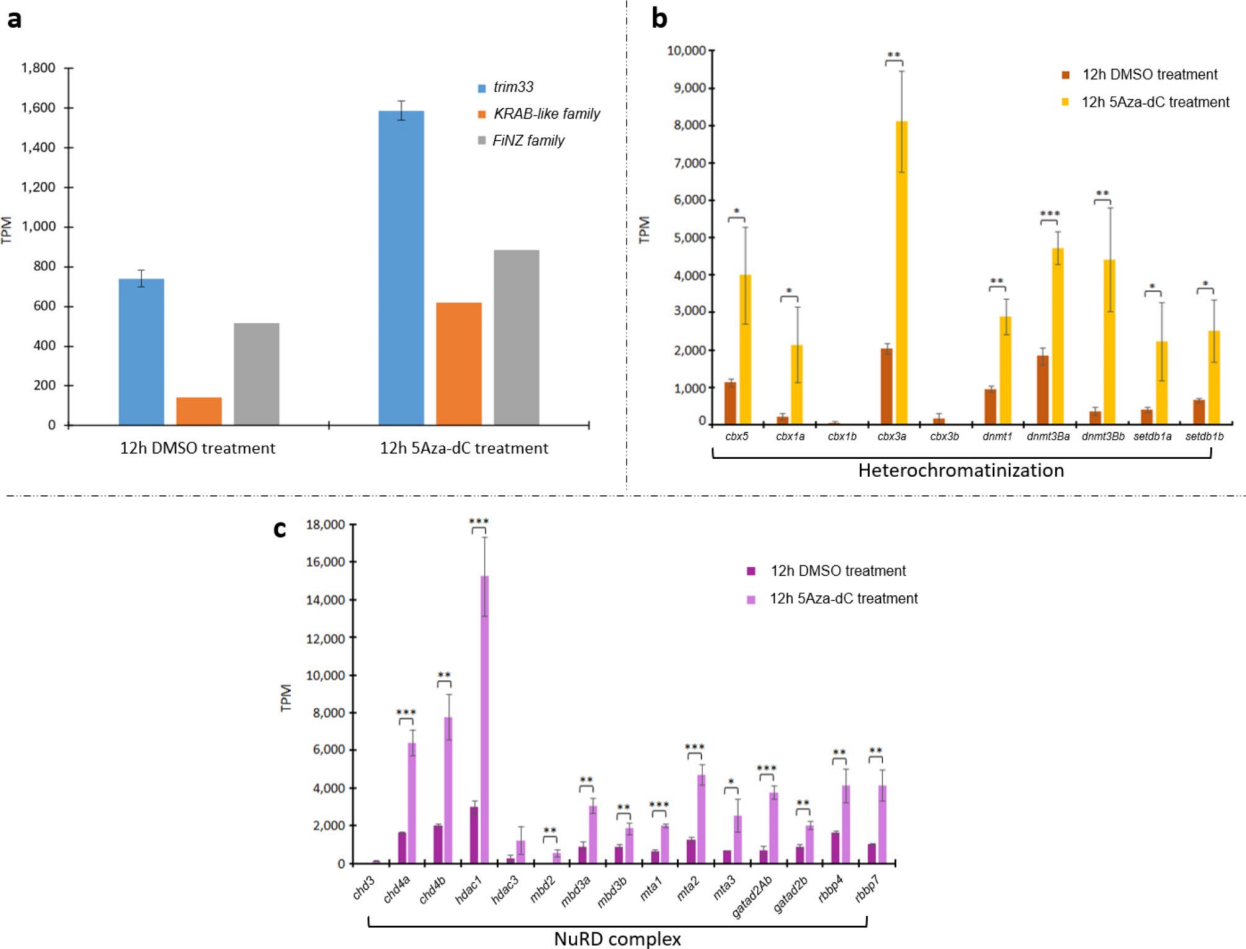
Expression of genes involved in TE silencing mechanisms in the RNA-seq data of 12 hpf zebrafish embryos treated with DMSO (used for control group) and 5Aza-dC. (**a**) Expression values of genes encoding *trim33*, *KRAB-like family*, and *FiNZ family* in the RNA-seq data of 12 hpf zebrafish embryos treated with DMSO and 5Aza-dC. We used «family» to indicate that the expression values reported for *KRAB-like* and *FiNZ ZNF*s were an average of values related to different genes. (**b**) Expression values of genes encoding proteins involved in heterochromatin formation in the RNA-seq data of 12 hpf zebrafish embryos treated with DMSO and 5Aza-dC. The statistical analysis was performed using one-way ANOVA. Statistically significant differences are presented as * for *p* < 0.05, ** for *p* < 0.01, and *** for *p* < 0.001. (**c**) Expression values of genes encoding proteins forming the NuRD complex in the RNA-seq data of 12 hpf zebrafish embryos treated with DMSO and 5Aza-dC. The statistical analysis was performed using one-way ANOVA. Statistically significant differences are presented as * for *p* < 0.05, ** for *p* < 0.01, and *** for *p* < 0.001.

To verify the response of genes involved in the TE silencing mechanism investigated here, the available RNA-Seq data of zebrafish embryos at 12 hpf treated with 5-aza-2’-deoxycytidine (5Aza-dC) were explored^17^. Upon treatment with this DNA methyltransferase inhibitor, TEs were derepressed, resulting in an increase in transcription levels (Fig. 6a). The major impact was due to LTR retroelements (Fig. 6b), particularly DIRS and ERV1 and 2 (Fig. 6c). TE derepression led to a general increase in the transcriptional activity of genes involved in their silencing/control (Fig. 7), which supports our hypothesis of their involvement in TE regulation. Since Meng and colleagues^17^ reported both up- and downregulation of gene expression levels after 5Aza-dC treatment, the upregulation of target genes might not be explained by general hypomethylation after DNMT inhibition but was likely associated with the necessity of counteracting TE activation. Moreover, Kimura distance-based copy divergence analyses revealed that activated LTR elements are mainly young copies (Fig. 6d; Fig. S5). Overall, these findings suggest that when LTRs are highly expressed, their regulators are also transcribed.

### Phylogenetic analysis of the PRDM9, KRAB, KRAB-like, and FiNZ proteins

**Fig. 8:**
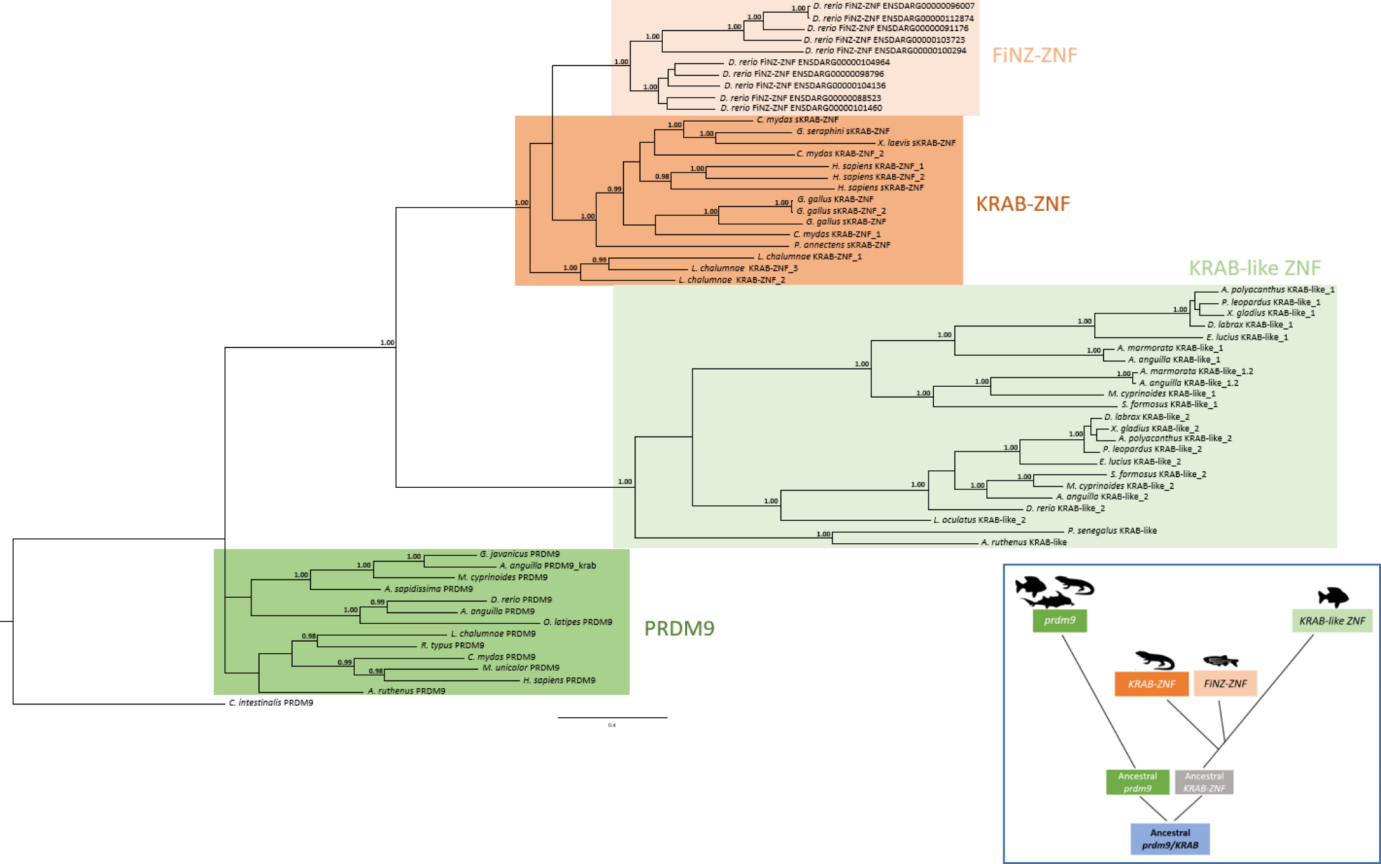
Phylogenetic analysis of PRDM9, KRAB, KRAB-like, and FiNZ proteins. Phylogenetic tree of PRDM9, KRAB, KRAB-like, and FiNZ amino acid sequences. Bayesian inference: 2,000,000 generations, sampling every 100, Jones substitution model, stationarity defined as when the average standard deviation of split frequencies approaching 0.0048, burn-in set to 2,500. Number besides notes indicates posterior probability values (> 0.97). Colored boxes group ortholog sequences corresponding to the proteins involved in the phylogenetic analysis. In the blue box, schematic representation of *prdm9*, *KRAB*, *KRAB-like*, and *FiNZ ZNFs* evolutionary history in gnathostomes.

The relationships of these Krüppel ZFPs were investigated through phylogenetic analyses to elucidate their evolutionary history (Fig. 8). Sequences belonging to PRDM9/Meisetz were also considered since they have an ancestral KRAB domain at the N-terminus that is considered an ancestor of sarcopterygian KRAB ZFPs^32^.

In the external position, the obtained tree revealed sequences belonging to PRDM9 even if they did not form a unique clade. This could be explained by the high degree of sequence divergence that may have accumulated compared with the PRDM9 ancestor sequence. In addition, the tree topology revealed two clades: one including sarcopterygian KRAB ZFPs and *D. rerio* FZFPs and the other comprising actinopterygian KRAB-like ZFPs. This scenario together with the other findings presented here suggest the evolutionary history shown in Fig. 8. The presence of a unique gene (*PRDM9*) in the urochordate *Ciona intestinalis* and in the cartilaginous fish *Rhincodon typus* suggested that a first duplication event occurred in the common ancestor of Osteichthyes (clade including ray-finned fish and lobe-finned fish), resulting in an ancestral *KRAB ZNF* gene. This gene may have given rise to the *KRAB ZNFs* of sarcopterygians and the *KRAB*-*like ZNFs* of actinopterygians. Although the KRAB-like domain has greater similarity to the KRAB domain (Fig. 8), the longer branches of KRAB-like ZNFs indicate greater divergence of these sequences than the KRAB ZNFs in relation to the PRDM9 sequences, mainly due to differences in the zinc finger sequences. In Cypriniformes, lineage-specific duplication event led to *FiNZ ZNFs* in addition to *KRAB-like* ZNFs^16^. The closer relationship of FZNFs with sarcopterygian KRAB ZNFs shown in the phylogenetic tree is due to the major similarity of C-terminal zinc finger motifs. Overall, our results suggest a common origin of the KRAB, KRAB-like, and FiNZ domains and that their related genes experienced lineage-specific expansions.

## Conclusions

Our data suggest the involvement of KRAB-like/TRIM33 proteins in TE control in actinopterygians, similar to KRAB/TRIM28 in sarcopterygians. These findings could lay a new foundation for understanding the evolutionary mechanisms underlying the adaptation of such diverse evolutionary lineages^33–35^. Moreover, as ray-finned fish are strictly influenced by abiotic factors, TEs and their controlling/silencing machinery potentially represent their advantages in coping with environmental disruptions that occur due to global change in the Anthropocene era.

## Materials and methods

### Superimposition of 3D protein structures

The structural predictions of eel KRAB-like and zebrafish FiNZ domains were obtained with the online server Iterative Threading ASSEmbly Refinement (i-tasser)^36^. The retrieved 3D protein structures were superimposed with the KRAB domain of the human KZNF93 downloaded from the PDB protein database^37^ via the MatchMaker tool from UCSF Chimera v1.12^38^. The same procedure was applied to the RBCC domains of human TRIM28 and TRIM33 downloaded from protein databases^39,40^ and the RBCC domains of human TRIM33 and zebrafish TRIM33 downloaded from AlphaFold^41^ (Supplementary Fig. S1).

### Plasmid design, transfection and coimmunoprecipitation

The original plasmids were ordered at GeneArt Gene Synthesis, and the inserts were cloned and inserted into the destination vector pTRE3HA or pTRE-FLAG via Gateway LR cloning, which expressed proteins with C-terminal HA and N-terminal FLAG tags (in a doxycycline-dependent manner).

293T cells (7.5 × 10^6^) were transfected with 6 µg of plasmid DNA with FuGENE6 (Promega) transfection reagent according to the manufacturer’s protocol. For HA- and FLAG-tagged constructs, doxycycline was added at a concentration of 1 µg/ml for 20 h during transfection.

After 20 h of transfection, the cells were washed twice with ice-cold PBS and incubated for 30 min on ice with gentle agitation in immunoprecipitation buffer (500 mM Tris HCl pH 7.4, 20 mM EDTA, 40 mM NaF, 30 mM sodium pyrophosphate decahydrate, 2 mM benzamidine, 1 mM PMSF, protease inhibitors and 0.5% NP40). The samples were then collected and sonicated three times via a probe sonicator (10 s, 30% amplitude). Cellular debris was removed by centrifugation at 5000 rpm for 5 min. The samples were incubated overnight on a rotating wheel at 4 °C with 50 µl of Pierce Anti-DYKDDDDK (FLAG) agarose magnetic beads (Thermo Fisher, A36797). The samples were then centrifuged at 5000 rpm for 5 min and washed three times with immunoprecipitation buffer with 0.05% NP40 on ice. The immunoprecipitates were then eluted in 50 µl, subjected to SDS‒PAGE, and analysed by immunoblotting with HRP-conjugated anti-HA (1:1000, Biolegend, clone 16B12) and anti-FLAG (1:1000, Invitrogen, MA1-91878-HRP) antibodies diluted in PBS supplemented with 0.1% Tween 20 and 5% (m/v) milk.

## Analyses of zebrafish developmental RNA-seq data

Triplicates of raw RNA-seq data from six different developmental stages (zygote, blastula, gastrula, somitogenesis, pharyngula, and larvae) and triplicates of two sets of RNA-seq data from 12 hpf zebrafish embryos treated with DMSO and 5Aza-dC were obtained from the Sequence Read Archive (SRA)^42^ under the accession numbers reported in supplementary table S1^17,26^. Raw paired-end reads (100 bp long for the dataset related to zebrafish development and 150 bp long for the dataset related to 12 hpf-treated embryos) were trimmed, and *de novo* assembly of the transcriptomes was performed with CLC Genomics Workbench v.12 (Qiagen, Hilden, Germany).

## Estimation of TE transcriptional activity and Kimura distance-based TE age distribution

To estimate TE transcriptional activity, we first identified TEs in the *de novo* assembled transcriptomes with RepeatMasker v.4.1.0^43^ via a specific zebrafish TE library downloaded from the FishTEDB database^44,45^. After TE identification, the trimmed reads related to six different developmental stages and two RNA-seq datasets of 12 hpf zebrafish embryos treated with DMSO and 5Aza-dC were mapped against the reference transcriptome via the proprietary map reads to the reference tool included in the CLC Genomics Workbench v.24.0.1 (Qiagen, Hilden, Germany), with the following mapping parameters: length fraction = 0.80 and similarity fraction = 0.80. From the RepeatMasker output file, redundancy was limited by removing the entries not classified as TEs and filtering for the highest score and length value (> 80 bp). For each TE type (LINE, SINE, LTR, DNA transposons, and unclear), the expression values were summed and then transformed in percentage of mapped reads relative to the total number of reads mapped to the reference transcriptome assemblies in each sample to achieve comparability between conditions^31^.

To assess the TE age distribution, the TE entries with the highest score and length values were extracted with Strawberry. The scripts “calcDivergenceFromAlign.pl” and “createRepeatLandscape.pl” provided by the RepeatMasker package were used to obtain Kimura distance (rate of transition and transversions) landscapes.

### Identification and expression analyses of genes involved in TE silencing

Genes of interest were searched through TBLASTN^46^ in the RNA-seq data considered. In particular, the gene set included: for heterochromatinization *chromobox homolog 5* (*cbx5*), *chromobox homolog 1a* (*cbx1a*), *chromobox homolog 1b* (*cbx1b*), *chromobox homolog 3a* (*cbx3a*), *chromobox homolog 3b* (*cbx3b*), *DNA (cytosine-5-)-methyltransferase 1* (*dnmt1*), *DNA (cytosine-5-)-methyltransferase 3 beta a* (*dnmt3βa*), *DNA (cytosine-5-)-methyltransferase 3 beta b* (*dnmt3βb*), *SET domain bifurcated histone lysine methyltransferase 1a* (*setdb1a*) and *SET domain bifurcated histone lysine methyltransferase 1b* (*setdb1b*); for NuRD complex *chromodomain helicase DNA binding protein 3* (*chd3*), *chromodomain helicase DNA binding protein 4a* (*chd4a*), *chromodomain helicase DNA binding protein 4b* (*chd4b*), *histone deacetylase 1* (*hdac1*), *histone deacetylase 3* (*hdac3*), *methyl-CpG binding domain protein 2* (*mbd2*), *methyl-CpG binding domain protein 3a* (*mbd3a*), *methyl-CpG binding domain protein 3b* (*mbd3b*), *metastasis associated 1* (*mta1*), *metastasis associated 1 family*,* member 2* (*mta2*), *metastasis associated 1 family*,* member 3* (*mta3*), *GATA zinc finger domain containing 2ab* (*gatad2ab*), *GATA zinc finger domain containing 2b* (*gatad2b*), *retinoblastoma binding protein 4* (*rbbp4*), and *retinoblastoma binding protein 7* (*rbbp7*). Both genes involved in heterochromatinization and those belonging to the NuRD complex were considered given their role in creating transcriptionally repressed chromatin at the TE sequence level. In addition, *trim33*, *KRAB-like ZNFs*, and *FiNZ ZNFs* were searched in all assembled transcriptomes. *KRAB-like ZNFs* are specific to actinopterygians^15^, whereas *FiNZ ZNFs* are specific to cyprinids^16^. Genes showing sequence similarity were searched, and their orthology was assessed through phylogenetic analysis (see the Phylogenetic analyses section for more details). These ZNF proteins were chosen because of their involvement in TE control^15,16^. The values of transcriptional activity were calculated following the workflow described in our previous work^47^. Briefly, trimmed sequencing reads obtained from each replicate of each stage/condition were mapped against the transcriptome via the RNA-seq mapping tool of the CLC Genomics Workbench v.24.0.1 (Qiagen, Hilden, Germany), with the following mapping parameters: length fraction = 0.80 and similarity fraction = 0.80. The gene expression values reported as transcripts per million (TPM) values were calculated via a scaling factor obtained from the cumulative expression of 2,111 single-copy orthologues shared by all the conditions analysed.

The Pearson correlation coefficient was calculated to assess the relationships between the expression values of TEs and genes of interest. To support these analyses, the Pearson correlation coefficient was evaluated using a null dataset of genes randomly chosen (Supplementary Table S2).

For the RNA-seq data of 12 hpf zebrafish embryos treated with DMSO and 5Aza-dC, the gene expression and TE transcriptional activity data obtained from the three replicates are expressed as the means ± standard errors, and statistically significant differences were evaluated via one-way ANOVA. The symbol * indicates p values < 0.05, ** indicates p values < 0.01, and *** indicates p values < 0.001.

### Phylogenetic analyses

Phylogenetic analyses were carried out to better understand the evolutionary history of genes of interest and verify the orthology of KRAB-like ZNF sequences (Supplementary Fig. S6) expressed in the RNA-seq data considered. The sequences were downloaded from NCBI GenBank^48^ or ENSEMBL^49^ (Supplementary Table S3), and multiple alignments were performed via CLUSTALW^50^ with default parameters. The amino acid substitution model and phylogenetic trees were obtained via MrBayes (version 3.2).

## Electronic supplementary material

Below is the link to the electronic supplementary material.

Supplementary Material 1

## Data Availability

RNA-seq data analysed during this study were obtained from the Sequence Read Archive (SRA)(42) under the accession numbers reported in supplementary table S1(17,26).
