# Supplementary material for: The arms race of ray-finned fish against the derepression of LTR retroelements
Source: Sci Rep. 2024 Nov 27;14:29431. doi: 10.1038/s41598-024-81149-9 (PMC11603059; doi:10.1038/s41598-024-81149-9)
Supplement: Supplementary file 1 — Supplementary Material 1 [file 41598_2024_81149_MOESM1_ESM.pdf]

Supplementary Material

The arms race of ray-finned fish against the derepression of LTR retroelements

Carotti Elisa†, Tittarelli Edith†, Carducci Federica, Barucca Marco*, Canapa Adriana, Biscotti Maria Assunta.

Dipartimento di Scienze della Vita e dell'Ambiente, Università Politecnica delle Marche, Via Brecce Bianche, 60131, Ancona (Italy).

*Corresponding author e-mail: m.barucca@univpm.it

† These authors equally contributed.

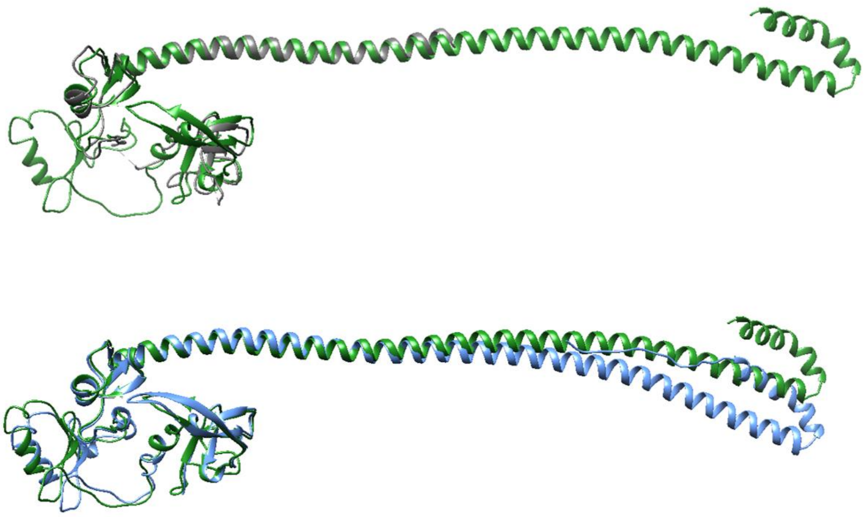

Supplementary Figure S1. Superimposition of 3D structure of *D. rerio* TRIM33 and that of *Homo sapiens* TRIM28. In the upper side the superimposition of TRIM28 RBCC domain in grey with TRIM33 RBCC domain in green in *Homo sapiens.* In the lower side superimposition of *Homo sapiens* TRIM33 RBCC domain in green and *Danio rerio* TRIM33 RBCC domain in light blue.

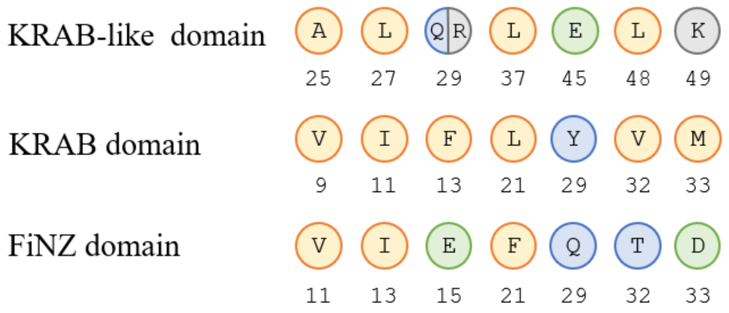

Supplementary Figure S2. Comparison between KRAB-like, KRAB, and FiNZ domains. In orange hydrophobic residues; in green acid residues; in blue hydrophilic residues; in grey basic residues. Numbers indicate the amino acid position.

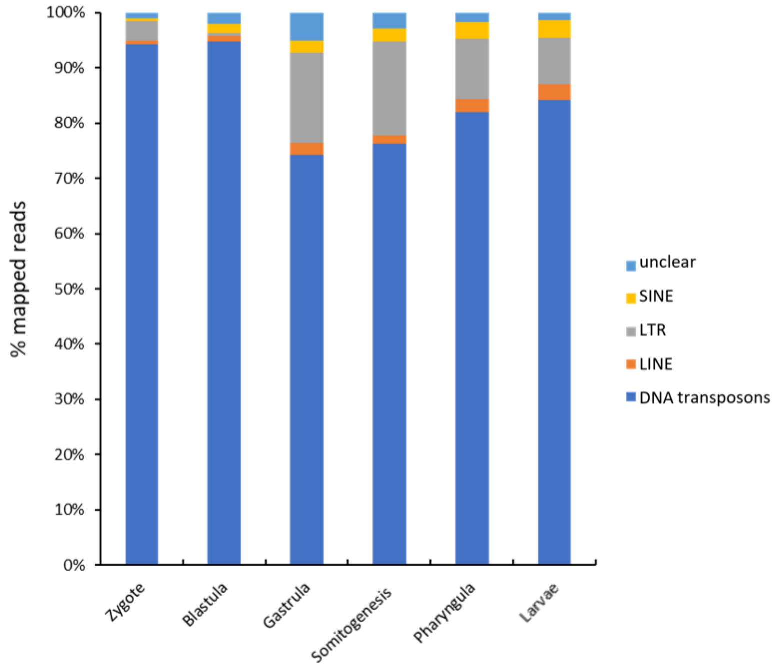

Supplementary Figure S3. Relative abundance of TE types. The relative abundance of the percentage mapped TE reads in six zebrafish developmental stages is reported.

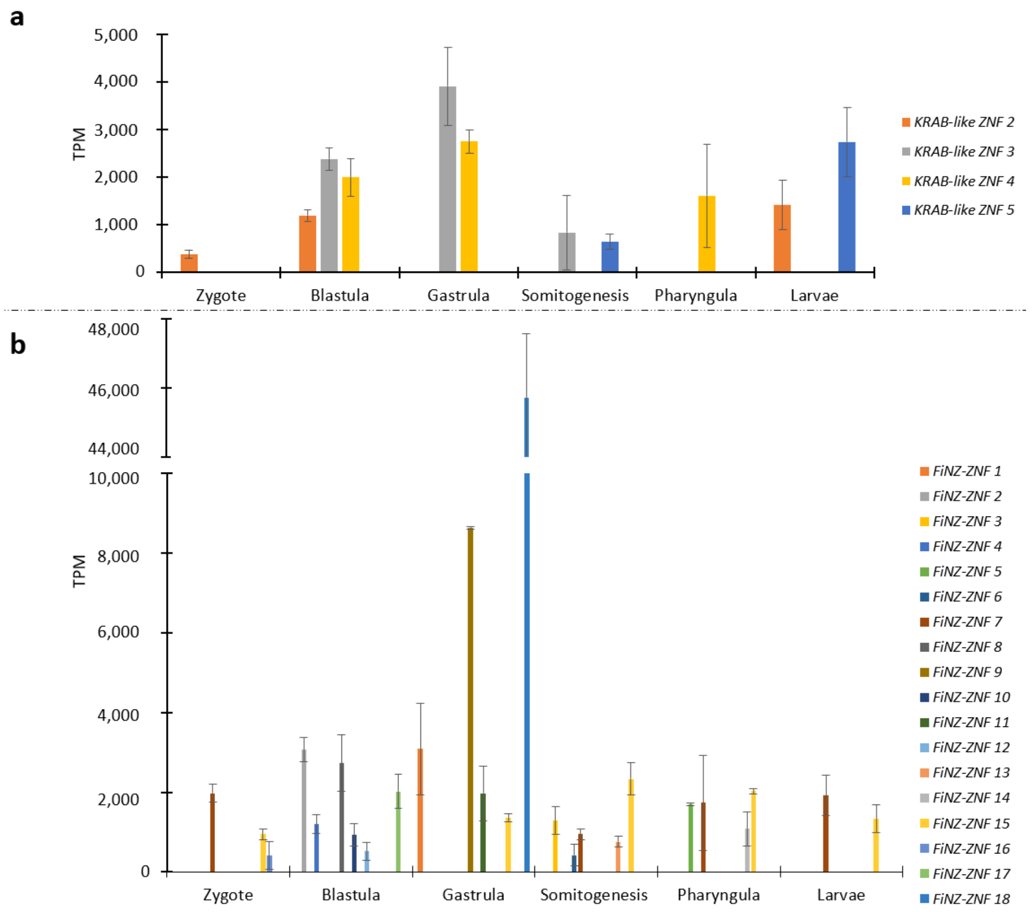

Supplementary Figure S4. Expression values of genes encoding KRAB-like and FiNZ ZFPs in six zebrafish developmental stages a. Expression values of genes encoding KRAB-like ZFPs in zebrafish developmental stages is reported. b. Expression values of genes encoding FiNZ ZFPs in zebrafish developmental stages.

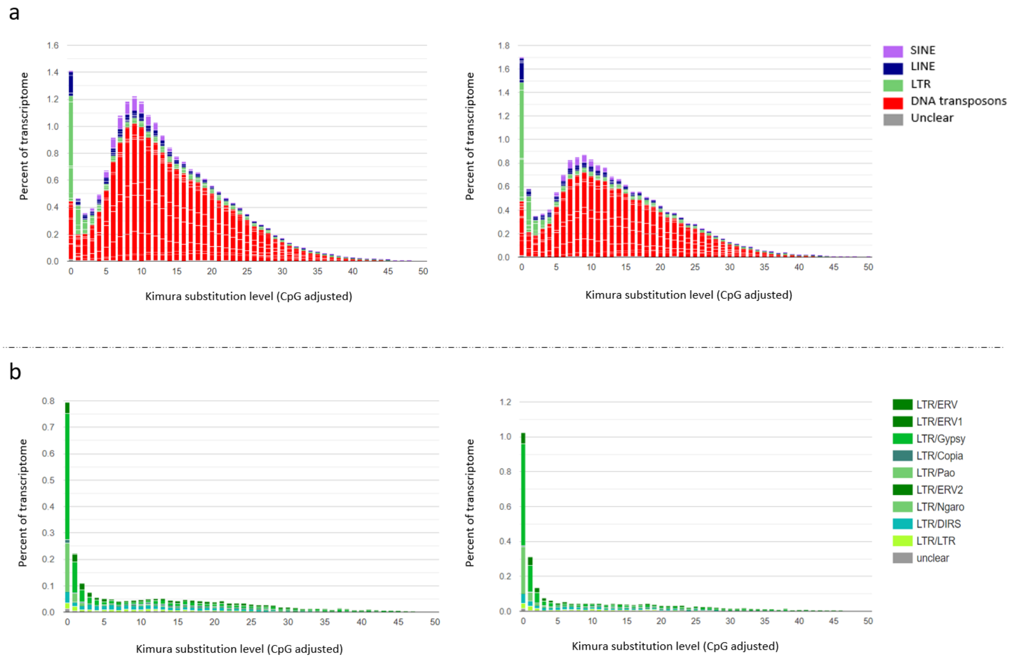

Supplementary Figure S5. A. TE repeat landscape plot. a. The repeat landscape plot obtained by Kimura distance-based copy divergence analyses of TEs in the RNA-seq data of 12 hpf zebrafish embryos treated with DMSO and 5Aza-dC. b. The repeat landscape plot obtained by Kimura distance-based copy divergence analyses of LTR retroelements in the RNA-seq data of 12 hpf zebrafish embryos treated with DMSO and 5Aza-dC.

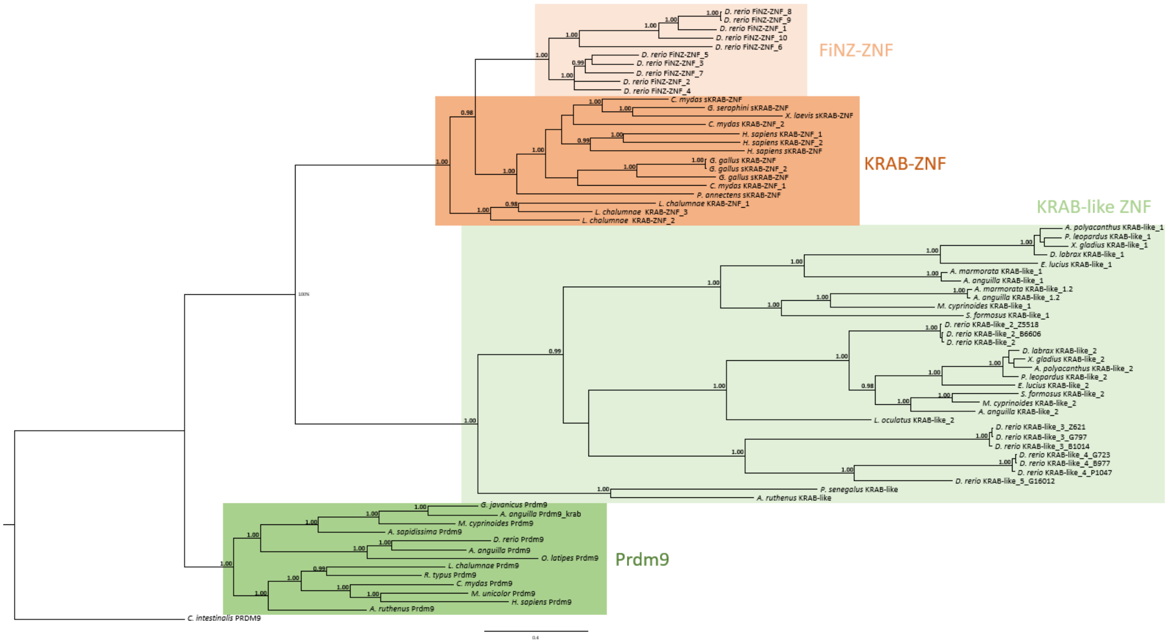

Supplementary Figure S6. Phylogenetic analysis of PRDM9, KRAB, KRAB-like, and FiNZ proteins. Phylogenetic tree of PRDM9, KRAB, KRAB-like, and FiNZ amino acid sequences. Bayesian inference: 2,000,000 generations, sampling every 100, Jones substitution model, stationarity defined as when the average standard deviation of split frequencies approaching 0.0023, burn-in set to 2,500. Colored boxes group ortholog sequences corresponding to the proteins involved in the phylogenetic analysis.

Accession number	Period	Stage	hpf	Treatment
ERR1442646<br>ERR1442647<br>ERR1442648	Zygote	1 cell	0	/
ERR1442631<br>ERR1442632<br>ERR1442633	Blastula	128 cell	2.25	/
ERR1442561<br>ERR1442562<br>ERR1442563	Gastrula	50% epiboly	5.25	/
ERR1442611<br>ERR1442612<br>ERR1442613	Somitogenesis	14-19 somites	16	/
ERR1442571<br>ERR1442572<br>ERR1442573	Pharyngula	Prim-15	30	/
ERR1442591<br>ERR1442592<br>ERR1442593	Larvae	Day 4	96	/
SRR18090815<br>SRR18090816<br>SRR18090817	Somitogenesis	~ 5-9 somites	12	DMSO
SRR18090812<br>SRR18090813<br>SRR18090814	Somitogenesis	~ 5-9 somites	12	5Aza-dC

Supplementary Table S1. Accession numbers of RNA-seq data analyzed and related zebrafish developmental stages and treatments.

		Pearson Correlation	P(T<=t) two tails
COX assembly mitochondrial protein (CMC2)	LTR	-0,19	0,14
	LINE	0,47	0,14
	SINE	0,46	0,14
	DNA transposons	0,04	0,14
	KRAB-like family	0,10	0,68
	FiNZ-ZNF family	-0,33	0,41

		Pearson Correlation	P(T<=t) two tails
Cytochrome b5 reductase 4 (CYB5R4)	LTR	-0,66	0,11
	LINE	-0,21	0,11
	SINE	-0,27	0,11
	DNA transposons	-0,47	0,12
	KRAB-like family	0,07	0,09
	FiNZ-ZNF family	-0,35	0,22

		Pearson Correlation	P(T<=t) two tails
Isopentenyl-diphosphate Delta-isomerase 1 (IDI1)	LTR	0,56	0,30
	LINE	0,02	0,30
	SINE	0,27	0,30
	DNA transposons	0,31	0,31
	KRAB-like family	-0,52	0,59
	FiNZ-ZNF family	-0,27	0,35

		Pearson Correlation	P(T<=t) two tails
Cyclin-H (CCNH)	LTR	0,37	0,10
	LINE	0,02	0,10
	SINE	-0,11	0,10
	DNA transposons	-0,16	0,10
	KRAB-like family	0,68	0,63
	FiNZ-ZNF family	0,88	0,30

		Pearson Correlation	P(T<=t) two tails
Myeloid differentitiaion primary response protein (MYD88)	LTR	-0,36	0,06
	LINE	0,06	0,06
	SINE	0,07	0,06
	DNA transposons	-0,07	0,06

	**KRAB-like family**	-0,12	0,08
	**FiNZ-ZNF family**	-0,48	0,21

		Pearson Correlation	P(T<=t) two tails
Filamin B (FLNB)	**LTR**	0,60	0,05
	**LINE**	0,33	0,05
	**SINE**	0,52	0,05
	**DNA transposons**	0,10	0,05
	**KRAB-like family**	-0,04	0,42
	**FiNZ-ZNF family**	0,00	0,80

Supplementary Table S2. Pearson correlation analyses between the null gene dataset with TE, KRAB-like family, and FiNZ-ZNF family during zebrafish development. For each correlation test the Pearson coefficients and related statistically significant values were reported.

Gene	Specie	Accession number	Name
FiNZ-ZNF	*D. rerio*	ENSDARG00000091176	*D. rerio* FiNZ-ZNF_1
FiNZ-ZNF	*D. rerio*	ENSDARG00000088523_ENSDART00	*D. rerio* FiNZ-ZNF_2
FiNZ-ZNF	*D. rerio*	ENSDARG00000098796_ENSDART00	*D. rerio* FiNZ-ZNF_3
FiNZ-ZNF	*D. rerio*	ENSDARG00000101460_ENSDART00	*D. rerio* FiNZ-ZNF_4
FiNZ-ZNF	*D. rerio*	ENSDARG00000104964_ENSDART00	*D. rerio* FiNZ-ZNF_5
FiNZ-ZNF	*D. rerio*	ENSDARG00000100294_ENSDART00	*D. rerio* FiNZ-ZNF_6
FiNZ-ZNF	*D. rerio*	ENSDARG00000104136_ENSDART00	*D. rerio* FiNZ-ZNF_7
FiNZ-ZNF	*D. rerio*	ENSDARG00000096007_ENSDART00	*D. rerio* FiNZ-ZNF_8
FiNZ-ZNF	*D. rerio*	ENSDARG00000112874_ENSDART00	*D. rerio* FiNZ-ZNF_9
FiNZ-ZNF	*D. rerio*	ENSDARG00000103723_ENSDART00	*D. rerio* FiNZ-ZNF_10
KRAB-like	*A. polyacanthus*	XP_022059640.1	*A. polyacanthus* KRAB-like_1
KRAB-like	*D. labrax*	XP_051274890.1	*D. labrax* KRAB-like_1
KRAB-like	*P. leopardus*	XP_042349212.1	*P. leopardus* KRAB-like_1
KRAB-like	*X. gladius*	XP_039976386.1	*X. gladius* KRAB-like_1
KRAB-like	*E. lucius*	XP_010894051.1	*E. lucius* KRAB-like_1
KRAB-like	*A. marmorata*	contig_scf718001089	*A. marmorata* KRAB-like_1.1
KRAB-like	*A. anguilla*	KAG5846213.1	*A. anguilla* KRAB-like_1.1
KRAB-like	*A. marmorata*	WOH20848.1	*A. marmorata* KRAB-like_1.2
KRAB-like	*A. anguilla*	XP_03528152	*A. anguilla* KRAB-like_1.2
KRAB-like	*M. cyprinoides*	XP_036406771.1	*M. cyprinoides* KRAB-like_1
KRAB-like	*S. formosus*	XP_029107817.1	*S. formosus* KRAB-like_1

KRAB-like	*L. oculatus*	XP_015208915.1	*L. oculatus* KRAB-like
KRAB-like	*P. senegalus*	KAG2468658.1	*P. senegalus* KRAB-like
KRAB-like	*A. ruthenus*	XP_058882726.1	*A. ruthenus* KRAB-like
KRAB-like	*D. rerio*	XP_003200019.1	*D. rerio* KRAB-like_1
KRAB-like	*D. labrax*	XP_051274889.1	*D. labrax* KRAB-like_2
KRAB-like	*A. anguilla*	KAG5846208.1	*A. anguilla* KRAB-like_2
KRAB-like	*S. formosus*	XP_029108239.1	*S. formosus* KRAB-like_2
KRAB-like	*M. cyprinoides*	XP_036406762.1	*M. cyprinoides* KRAB-like_2
KRAB-like	*E. lucius*	XP_010900134.1	*E. lucius* KRAB-like_2
KRAB-like	*X. gladius*	XP_039976512.1	*X. gladius* KRAB-like_2
KRAB-like	*A. polyacanthus*	XP_022078520.2	*A. polyacanthus* KRAB-like
KRAB-like	*P. leopardus*	XP_042349848.1	*P. leopardus* KRAB-like
KRAB-like	*D. rerio*	Zygote_5518	*D. rerio* KRAB-like_2_Z5518
KRAB-like	*D. rerio*	Zygote_621	*D. rerio* KRAB-like_3_Z621
KRAB-like	*D. rerio*	Blastula_6606	*D. rerio* KRAB-like_2_B6606
KRAB-like	*D. rerio*	Blastula_1014	*D. rerio* KRAB-like_3_B1014
KRAB-like	*D. rerio*	Blastula_977	*D. rerio* KRAB-like_4_B977
KRAB-like	*D. rerio*	Gastrula_797	*D. rerio* KRAB-like_3_G797
KRAB-like	*D. rerio*	Gastrula_16012	*D. rerio* KRAB-like_5_G16012
KRAB-like	*D. rerio*	Gastrula_723	*D. rerio* KRAB-like_4_G723
KRAB-like	*D. rerio*	Pharyngula_1047	*D. rerio* KRAB-like_4_P1047
KRAB-ZNF	*C. mydas*	XP_037743398.2	*C. mydas* KRAB-ZNF_1
KRAB-ZNF	*C. mydas*	XP_043384165.1	*C. mydas* KRAB-ZNF_2
KRAB-ZNF	*G. gallus*	XP_040505405.1	*G. gallus* KRAB-ZNF
KRAB-ZNF	*H. sapiens*	BAG62217.1	*H. sapiens* KRAB-ZNF
KRAB-ZNF	*H. sapiens*	AAI42997.1	*H. sapiens* KRAB-ZNF_2
KRAB-ZNF	*L. chalumnae*	XP_014352648.1	*L. chalumnae* KRAB-ZNF
KRAB-ZNF	*L. chalumnae*	XP_014339776.1	*L. chalumnae* KRAB-ZNF_2
KRAB-ZNF	*L. chalumnae*	XP_014343766.1	*L. chalumnae* KRAB-ZNF_3
Prdm9	*G. javanicus*	KAJ8281444.1	*G. javanicus* Prdm9
Prdm9	*M. cyprinoides*	XP_036372284.1	*M. cyprinoides* Prdm9
Prdm9	*A. sapidissima*	XP_041921253.1	*A. sapidissima* Prdm9
Prdm9	*A. ruthenus*	XP_058876519.1	*A. ruthenus* Prdm9
Prdm9	*L. chalumnae*	XP_005998057.1	*L. chalumnae* Prdm9
Prdm9	*C. mydas*	XP_007053470.2	*C. mydas* Prdm9
Prdm9	*M. unicolor*	XP_030069220.1	*M. unicolor* Prdm9
Prdm9	*H. sapiens*	NP_064612.2	*H. sapiens* Prdm9
Prdm9	*R. typus*	XP_020371194.1	*R. typus* Prdm9
Prdm9	*A. anguilla*	KAG5854928.1	*A. anguilla* Prdm9
Prdm9	*D. rerio*	NP_957196.1	*D. rerio* Prdm9
Prdm9	*O. latipes*	XP_011486653.1	*O. latipes* Prdm9
Prdm9	*C. intestinalis*	AK11	*C. intestinalis* Prdm9
Prdm9_KRAB	*A. anguilla*	KAG5849929.1	*A. anguilla* Prdm9KRAB
standard KRAB-ZNF	*C. mydas*	XP_043390044.1	*C. mydas* sKRAB-ZNF
standard KRAB-ZNF	*G. seraphini*	XP_033789429.1	*G. seraphini* sKRAB-ZNF
standard KRAB-ZNF	*X. laevis*	XP_041418209.1	*X. laevis* sKRAB-ZNF

standard KRAB-ZNF	*G. gallus*	XP_040509770.1	*G. gallus* sKRAB-ZNF_1
standard KRAB-ZNF	*G. gallus*	NP_001025866.2	*G. gallus* sKRAB-ZNF_2
standard KRAB-ZNF	*H. sapiens*	NP_001158187.1	*H. sapiens* sKRAB-ZNF_2
standard KRAB-ZNF	*P. annectens*	XP_043911432.1	*P. annectens* sKRAB-ZNF

Supplementary Table S3. Accession numbers of sequences used in phylogenetic analyses.

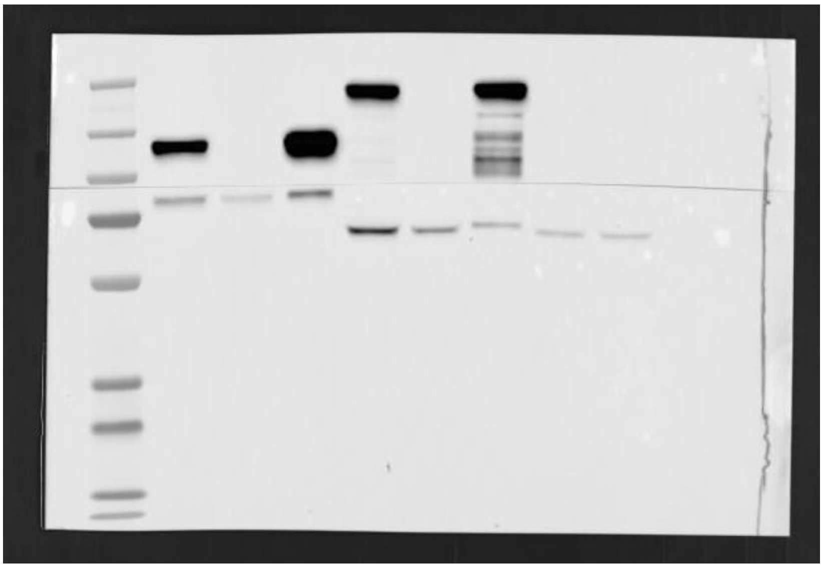

Full-length membrane blots referred to coIP assay after exposure time of 3 seconds and showed in Figure 1b in the main text.

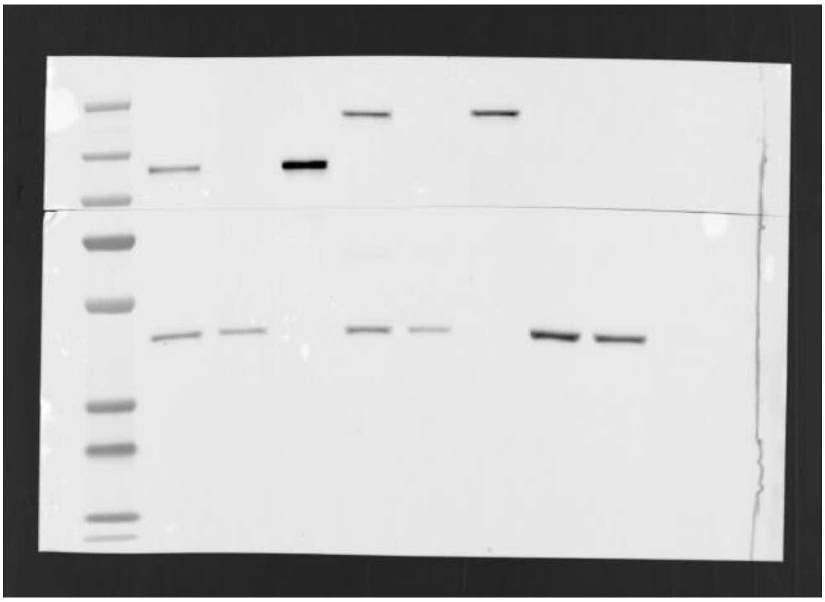

Full-length membrane blots referred to coIP assay after exposure time of 1 seconds and showed in Figure 1b in the main text.

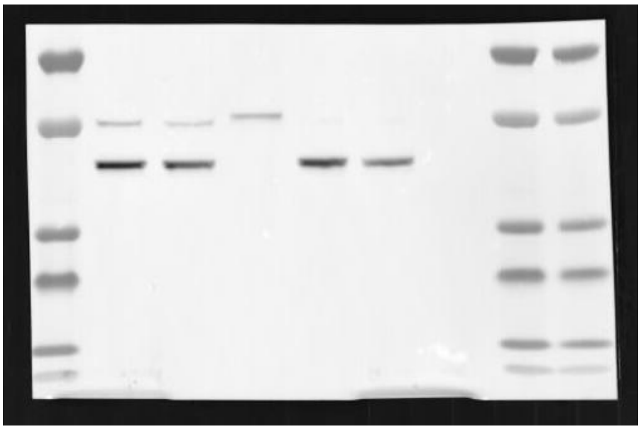

Full-length membrane blots referred to coIP assay after exposure time of 1 seconds and showed in Figure 1d in the main text.

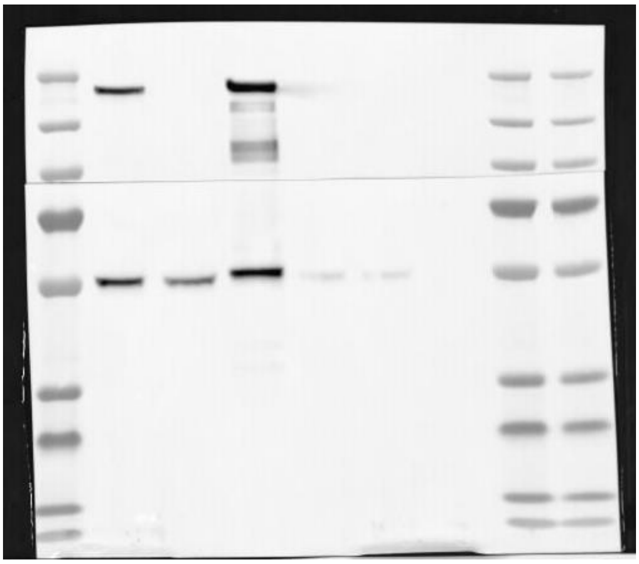

Full-length membrane blots referred to coIP assay after exposure time of 1 seconds and showed in Figure 1d in the main text.
